# Attosecond photoionisation time delays reveal the anisotropy of the molecular potential in the recoil frame

**DOI:** 10.1038/s41467-022-28783-x

**Published:** 2022-03-10

**Authors:** H. Ahmadi, E. Plésiat, M. Moioli, F. Frassetto, L. Poletto, P. Decleva, C. D. Schröter, T. Pfeifer, R. Moshammer, A. Palacios, F. Martin, G. Sansone

**Affiliations:** 1 Physikalisches Institut, Albert-Ludwigs-Universität, Stefan-Meier-Straße 19, 79104 Freiburg, Germany; 2 Dipartimento di Fisica, Politecnico di Milano, Piazza Leonardo da Vinci 32, 20133 Milano, Italy; 3 Universidad Autónoma de Madrid, Facultad de Ciencias Cantoblanco, Madrid, 28049 Spain; 4 Instituto Madrileño de Estudios Avanzados en Nanociencia (IMDEA-Nanociencia), Cantoblanco, 28049 Madrid, Spain; 5 CNR-IFN, Padua, Italy; 6 CNR IOM and Universitá di Trieste, 34127 Trieste, Italy; 7 Max-Planck-Institut für Kernphysik, 69117 Heidelberg, Germany; 8 Institute for Advanced Research in Chemical Sciences (IAdChem), Universidad Autónoma de Madrid, 28049 Madrid, Spain

**Keywords:** Optical techniques, Optical physics

## Abstract

Photoionisation time delays carry structural and dynamical information on the target system, including electronic correlation effects in atoms and molecules and electron transport properties at interfaces. In molecules, the electrostatic potential experienced by an outgoing electron depends on the emission direction, which should thus lead to anisotropic time delays. To isolate this effect, information on the orientation of the molecule at the photoionisation instant is required. Here we show how attosecond time delays reflect the anisotropic molecular potential landscape in CF_4_ molecules. The variations in the measured delays can be directly related to the different heights of the potential barriers that the outgoing electrons see in the vicinity of shape resonances. Our results indicate the possibility to investigate the spatial characteristics of the molecular potential by mapping attosecond photoionisation time delays in the recoil-frame.

## Abstract (web summary)

Photoionization time delays provide insights on the interaction of photon with the electrons. Here the authors explore the role of the molecular potential to the attosecond time delays in the photoionization of CF4 molecule.

## Introduction

Molecular systems are characterised by complex potential landscapes determined by their chemical composition and by the spatial arrangement of their constituents. In general, the electronic potential presents a non-spherical shape, which plays a key role in the stereo-dynamics of atom-molecule collisions^1^ and molecule–molecule interactions^2^.

As explained in textbooks, the effect and the spatial gradient of a potential can be unveiled by monitoring the motion of a probe charge immersed in that potential^3^. In atoms and molecules, this charge can be one of the electrons contained in the system, which must absorb enough energy from an external source to overcome the ionisation potential and to acquire the necessary kinetic energy to explore the potential landscape, while staying long enough in the molecular surroundings to sample its relevant features. This is ideally possible by using ultraviolet radiation, i.e. photon energies of a few tens eV. In a classical picture, an electron with 10 eV energy takes about 53 as to travel through the typical molecular extension of 1 Å. The extremely short timescale of this motion calls for the application of attosecond pulses, which can efficiently generate photoelectron wave packets and provide the necessary time resolution^4–7^.

The dynamics of photoionising wave packets is usually investigated by means of pump-probe experiments, in which an isolated or a train of attosecond pulses in the extreme ultraviolet (XUV) range set the photoelectron wave packet free and a synchronised infra-red (IR) pulse probes the instant at which the electron enters the continuum^8^. Using this approach, the role of electronic correlation effects in the photoionisation of atoms has been investigated in real-time^9–11^. Attosecond time delays have also been reported in photoionisation in molecular systems, showing the relevance of nuclear motion in hydrogen^12^ and the role played by shape resonances in N_2_O^13^ and nitrogen^14,15^. Moreover, the role of the localisation of the initial wave function^16^ and of a functional molecular group^17^ has also been demonstrated.

In atomic systems, the photoionisation time delays are usually decomposed into a term specific of the atomic potential (usually indicated as Eisenbud–Wigner–Smith delay^18^) and a measurement-induced contribution due to the action of the IR probe pulse on the photoelectron wave packet moving in the long-range Coulomb potential^19,20^. While in atoms the influence of the latter term can be usually quantified through simple formulas independent of the specific target^20^ and its angular dependence has been characterised^21^, in molecules the effect of the IR field on the measured time delays has not been characterised yet. In general, in the case of molecular systems, the contributions of the two terms cannot be disentangled^22^, which requires a more involved analysis.

A fundamental prerequisite for the characterisation of the combined effect of the anisotropic molecular landscape and of the IR field is to have access to the orientation of the molecule at the photoionisation instant. This can be done by measuring the emission direction(s) of ionic fragment(s) after the interaction with the XUV radiation, which defines the recoil frame. Symmetric molecules consisting of only a few atoms are ideal to test these effects in this frame. On the one hand, small molecules present a limited number of photoionisation and photofragmentation pathways, making feasible the identification of the electronic level of the outgoing photoelectron and, under suitable conditions, the determination of the molecular orientation during the interaction with the ionising radiation. On the other hand, the symmetry of the molecule gives the opportunity to identify specific privileged directions in the recoil frame and to characterise the effect of the molecular potential along them.

In this work we investigate the photoionisation dynamics induced by a train of attosecond XUV pulses on CF_4_ molecules by means of photoelectron-photoion coincidence spectroscopy^23^. The advantage of this approach is the possibility to derive information on the molecular orientation at the instant of photoionisation by measuring in coincidence the momenta of the emitted electron and the fragment ions resulting from the ulterior dissociation of the molecular cation^24^. In this way, we have been able to unambiguously identify individual ionisation channels and obtain time-resolved recoil-frame photoelectron angular distributions (RFPADs) from which the variations of photoionisation delays with the electron emission direction have been extracted. The measured delays are in very good agreement with those obtained from calculated time-resolved RFPADs where all the transitions induced by the attosecond pulse train and the IR probe, in particular those induced in the continuum by the IR pulse, are taken into account in a time-dependent formalism. The agreement confirms the validity of our experimental approach and opens the route to orientation-specific exploration and understanding of molecular photoionisation delays.

## Results

### Recoil frame and XUV spectroscopy of CF_4_

**Fig. 1:**
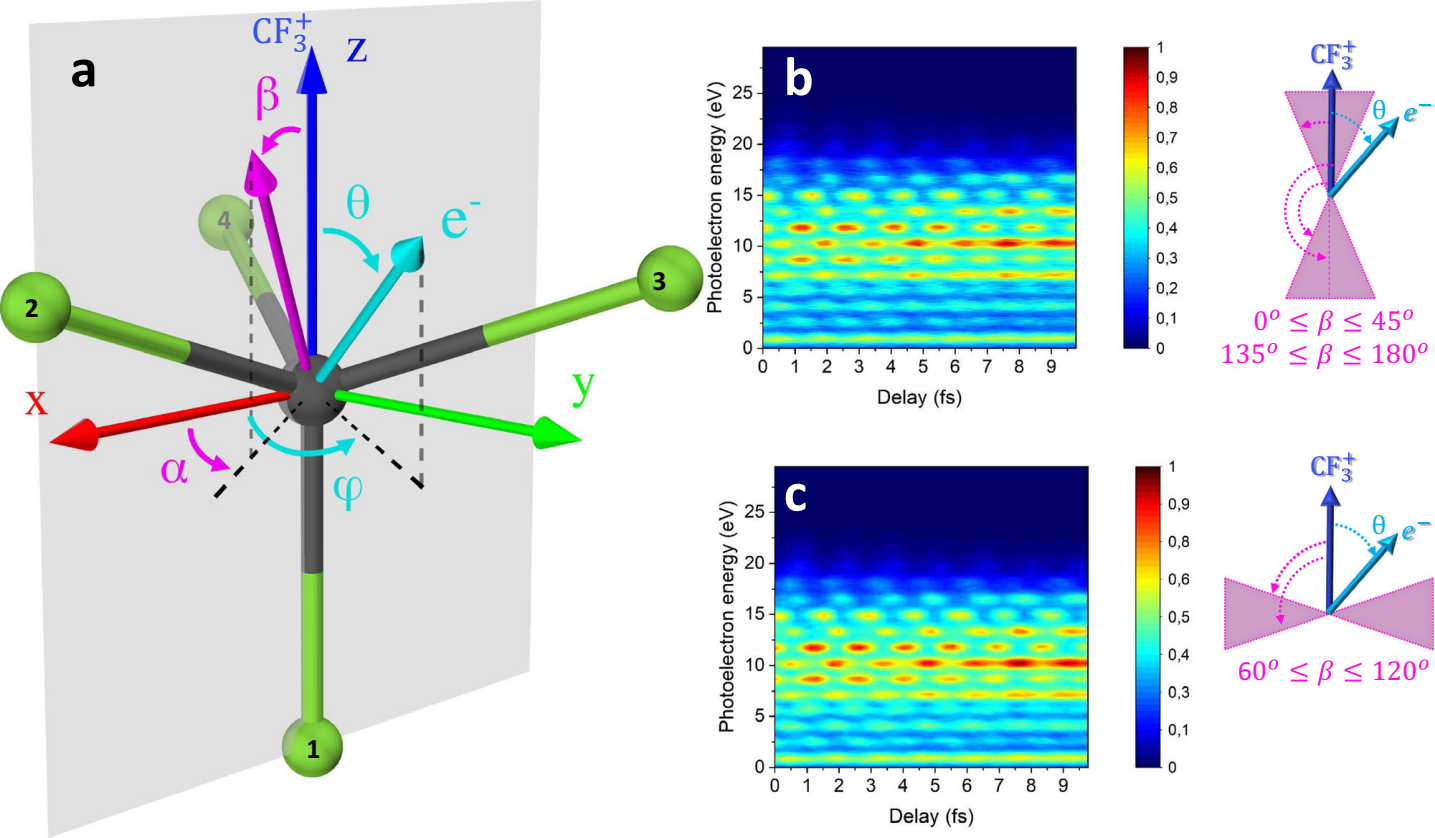
Molecular and recoil frames, and averaged RABBITT traces for the parallel and perpendicular cases. **a** CF_4_ molecule and definition of the different quantities in the molecular frame. The direction of emission of the CF${}_{3}^{+}$ ion defines the z-axis (blue arrow), which identifies the recoil frame in this experiment. The *x*-axis is contained in the plane identified by two fluorine atoms (1 and 2 in the figure). The orientation of the polarisation vector of the electric field (magenta arrow) is defined in the molecular frame by the angles *α* and *β*. The emission direction of the photoelectron in the molecular frame (cyan arrow) is defined by the angles *θ* and *φ*. RABBITT traces obtained for the parallel (0^∘^ ≤ *β* ≤ 45^∘^ and 135^∘^ ≤ *β* ≤ 180^∘^) **b** and perpendicular (60^∘^ ≤ *β* ≤ 120^∘^) **c** configurations. The angles *α* and *φ* cannot be determined in our measurements.

We focus on photoelectrons emitted from the Highest-Occupied Molecular Orbital of CF_4_, which is triply degenerate and belongs to the irreducible representation T_1_ of the point group T_*d*_ (see Fig. 1a). The xyz system represents the molecular frame, where one fluorine atom (1) is positioned along the negative z-axis and a second fluorine atom (2) is contained in the xz plane (the carbon atom occupies the centre). In the molecular frame, the common direction of the polarisation vectors of the collinear XUV and IR fields (indicated as a magenta arrow in Fig. 1a) is identified by the angles *β* (polar angle) and *α* (azimuthal angle).

Photoelectrons and photoions emitted after the interaction with an attosecond pulse train generated in argon and krypton and a synchronised IR field were measured using a Reaction Microscope^25^. This approach usually called RABBITT (Reconstruction of attosecond beating by two-photon transitions)^8^ allows one to combine temporal and spectral resolution, which turns out to be advantageous for identifying the different fragmentation channels or yields involved in the experiment. The setup used in the measurements is described in ref. ^26^.

For XUV photon energies in the range 20–46 eV photoionisation of neutral CF_4_ into five different cationic states (X^2^T_1_, A^2^T_2_, B^2^E, C^2^T_2_, D^2^A_1_) is energetically possible^27^. While the first three predominantly lead to the formation of CF${}_{3}^{+}$ ions, the last two lead to the formation of CF${}_{2}^{+}$ ions (see Supplementary Figs. 1 and 2 and Supplementary Table 1). The parent molecular ion CF${}_{4}^{+}$ was not observed, in agreement with previous spectroscopic measurements^28^. Additional correlation between the kinetic energy release (KER) of the ions and the photoelectrons gives the possibility to isolate the contribution of the photoelectrons associated with the X^2^T_1_ state from those associated with the A^2^T_2_ and B^2^E states (see Supplementary Figs. 3 and 4).

Photoionisation from the ground state (leading to cations in the X^2^T_1_ state) results in fast dissociation and emission of CF${}_{3}^{+}$ fragments with 100% probability^29^, giving access to the relative orientation of the polarisation direction of the external fields with respect to the polar angle *β* (the azimuthal angle *α* cannot be determined in our measurements) at the instant of photoionisation using the recoil approximation^30^. The validity of the approximation is further confirmed by the good agreement between the measured photoelectron angular distributions (PADs) for this state and those calculated by assuming a fixed nuclei configuration (see below). Because the position of the fluorine atoms in the tripod is not determined in the experiment, the measured PADs are plotted as a function of the *θ* angle, i.e. with respect to the z-axis (see Fig. 1), which defines the recoil frame and are therefore integrated over the *φ* angle.

### RABBITT measurements

The photoelectron spectra measured as a function of the delay between the XUV and IR pulses for specific molecular orientations with respect to the light polarisation vector of the collinear fields are presented in Fig. 1b, c. The spectra are thus retrieved capturing only photoelectrons in coincidence with the momentum of the dissociating CF${}_{3}^{+}$ ion being parallel (0^∘^ ≤ *β* ≤ 45^∘^ and 135^∘^ ≤ *β* ≤ 180^∘^) or perpendicular (60^∘^ ≤ *β* ≤ 120^∘^) with respect to the light polarisation vector. The signal is therefore obtained by integrating over all possible photoelectron emission directions, but for the specific molecular orientations with respect to the light as indicated in the right side insets of Fig. 1. The excellent quality of the traces offers the opportunity to investigate the oscillation of the sidebands for different photoemission directions *θ* in the recoil frame.

**Fig. 2:**
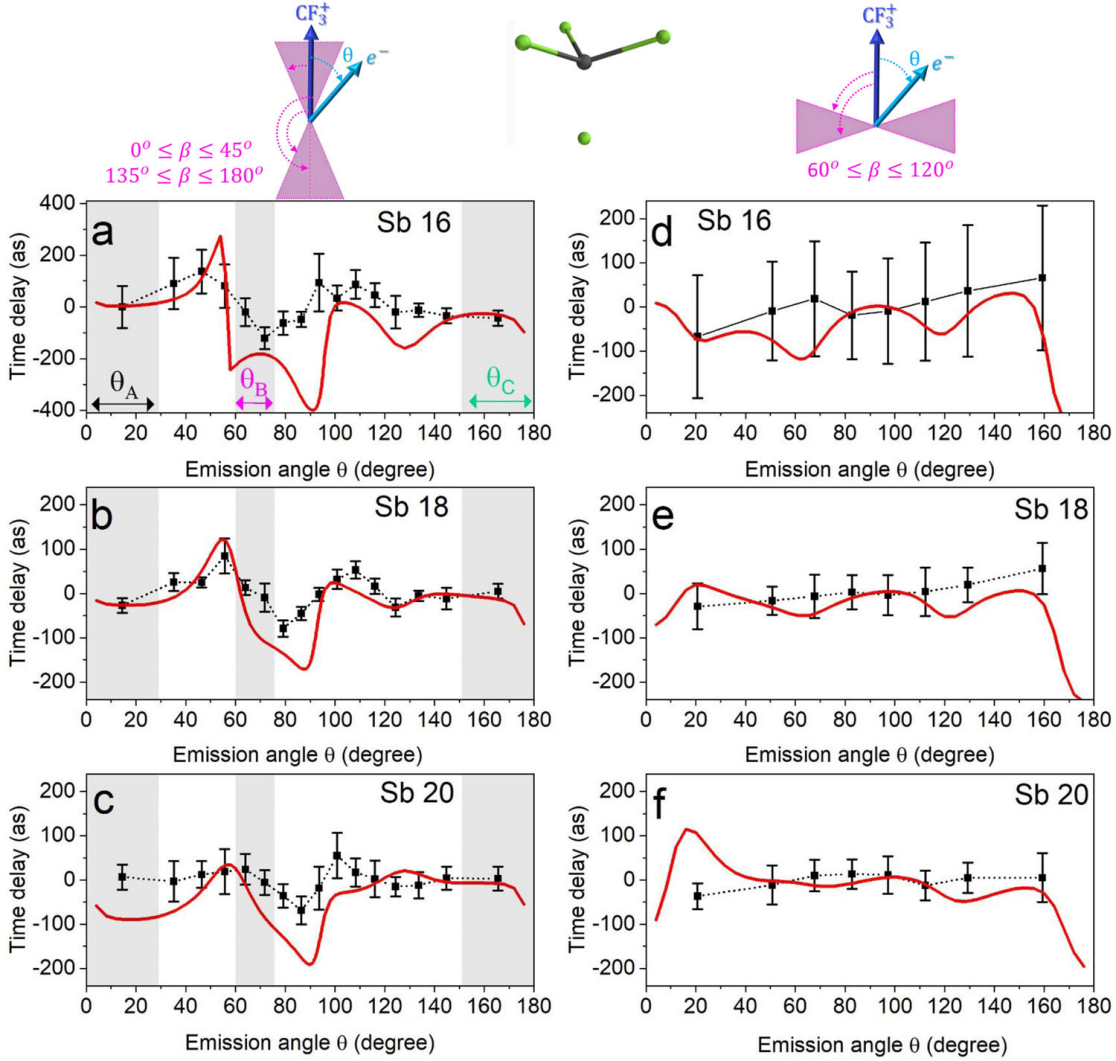
Experimental and simulated attosecond time delays for the parallel and perpendicular cases. Experimental (black points and black line) and theoretical (red lines) attosecond time delays as a function of the photoelectron emission angle *θ* along the recoil axis for the parallel (**a**–**c**) and perpendicular (**d**–**f**) configurations for the sidebands 16 (**a**, **d**), 18 (**b**, **e**), and 20 (**c**, **f**). The shaded areas in **a**–**c** indicate the three angle intervals *θ*_*A*_, *θ*_*B*_, and *θ*_*C*_. The error bars were obtained by weighting the photoionisation delays with the root-mean-square phase noise over an integrated electron kinetic-energy range of 1.2 eV centred around the maximum of each sideband^13^. The complete data set for all sidebands is presented in Supplementary Figs. 9 and 10.

The attosecond time delays determined for different recoil frame emission angles *θ* are reported in Fig. 2 for the parallel (panel a–c) and perpendicular (panel d–f) cases, respectively. The photoemission time delay averaged over the angle *θ* for the parallel and perpendicular cases were estimated from Fig. 1b, c and subtracted from the angular-resolved delays to remove the effect of the attosecond chirp (see also Supplementary Fig. 5).

### Theoretical modelling

The theoretical results obtained by solving the TDSE within the static-exchange DFT method described in^15,31^ and with laser parameters that reproduce the experimental conditions (see details in the SI), are also shown in Fig. 2. We checked that the simulations reproduce the photoelectron spectrum and the main features of the RFPADs generated by the XUV pulses for the parallel and perpendicular cases (see Supplementary Figs. 6–8). Considering the same fitting procedure as in the experiment, we have retrieved the time delays from the calculated RABBITT spectra for the parallel and perpendicular molecular orientations.

## Discussion

Overall the experimental and theoretical photoemission delays are in good agreement. The experimental attosecond time delays for the parallel case exhibit a minimum around *θ* = 90^∘^, while they are relatively independent of *θ* in the perpendicular case (see also Suppementary Figs. 9 and 10). The minima present negative values (on the order of a few hundreds of attoseconds), indicating that the maxima of the sidebands oscillations observed at these angles occur at earlier time delays. The theoretical data reproduce these trends, with the minimum at 90^∘^ for the parallel configuration and a smooth evolution as a function of the photoelectron emission angle for the perpendicular configuration.

**Fig. 3:**
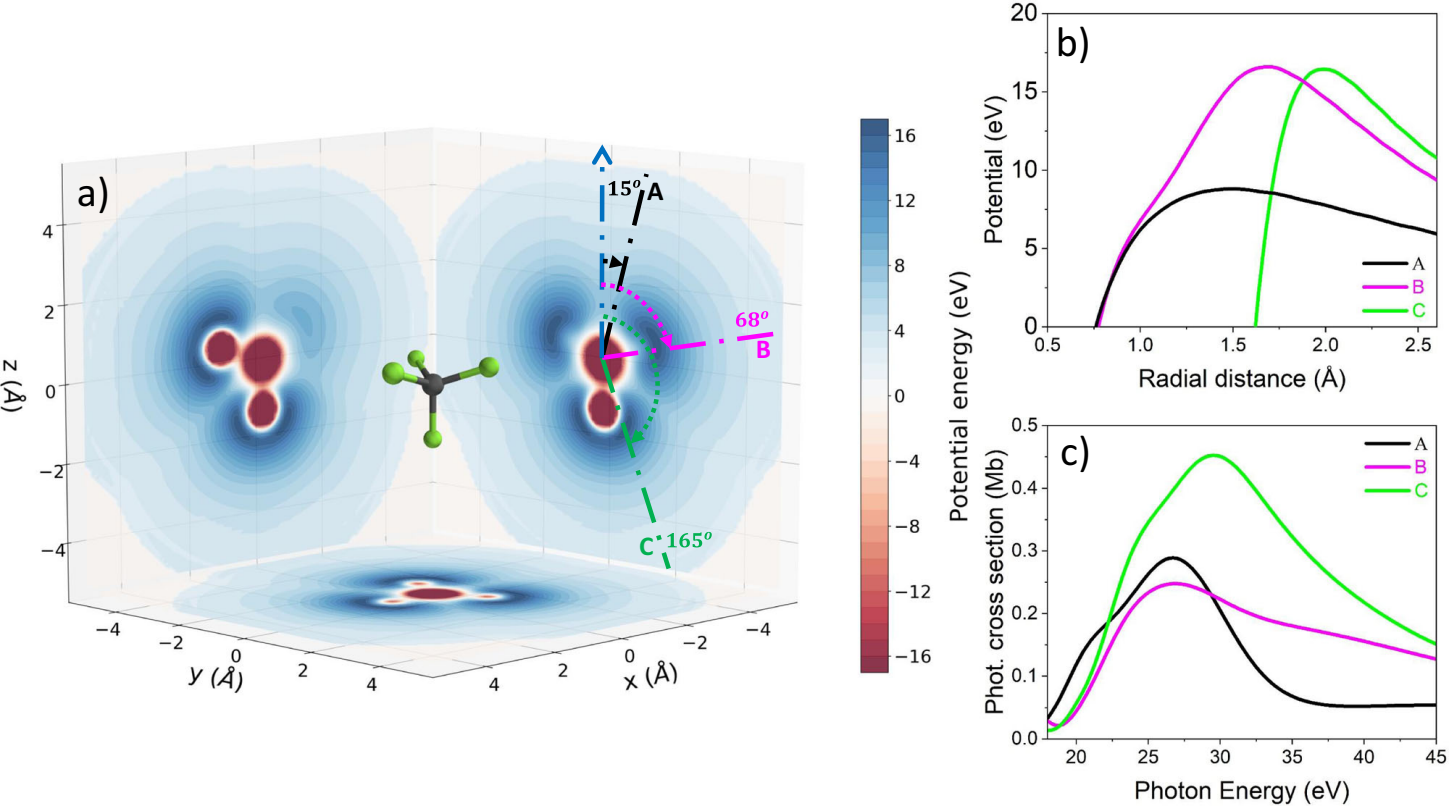
Molecular potential and photoionisation cross-sections. **a** Two-dimensional cuts of the molecular potential along the *xy*, *xz* and *yz* planes. **b** Molecular potential along the three directions indicated in **a**. **c** Photoionisation cross-sections along the three directions indicated in **a**.

For the parallel case (left panels in Fig. 2), the minimum observed around *θ* = 90^∘^ can be attributed to the interaction with the IR field (see Supplementary Figs. 11–13). In Fig. 2a–c, three different regions are highlighted corresponding to the intervals *θ*_*A*_ = [0^∘^ − 29^∘^], *θ*_*B*_ = [60^∘^ − 75. 5^∘^], and *θ*_*C*_ = [151^∘^ − 180^∘^] centred around the directions A (*θ* = 15^∘^), B (*θ* = 68^∘^) and C (*θ* = 165^∘^), respectively. Photoelectrons leaving the molecule along these three directions, experience significant differences in the molecular landscape, as shown in Fig. 3a, which reports the molecular potential resulting from the simulations and the three escape directions A, B, and C (indicated with dot-dashed lines). For a better visualisation, two-dimensional cuts of the potential are plotted in each plane. The nearly opposite directions A and C correspond to photoelectrons emitted (almost) parallel or antiparallel to the ion emission direction. For these directions, the molecular potentials exhibit barriers with quite different heights, as shown in Fig. 3b. On the contrary, the direction B corresponds to photoelectrons escaping the molecule along a direction characterised by a barrier, whose height is close to that along the C direction. The photoionisation cross-sections along the three directions are also reported in Fig. 3c, indicating the existence of shape-resonances in the photon-energy range ≈ 28-33 eV for the three directions.

**Fig. 4:**
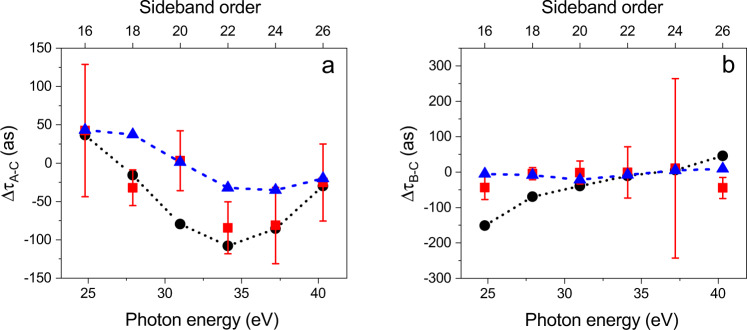
Influence of the anisotropic molecular potential on attosecond time delays. Difference of attosecond time delays between the emission directions A (*θ* = 15^∘^) and C (*θ* = 165^∘^) (**a**) and B (*θ* = 68^∘^) and C (**b**) for the experiment (red squares) and extracted from the simulations (black circles). The RABBITT spectra were previously averaged over the intervals of emission angles *θ*_*A*_, *θ*_*B*_, and *θ*_*C*_ for the emission directions A, B, and C, respectively. The blue triangles correspond to the differences of the stereo-Wigner time delays between the directions A and C (**a**) and B and C (**b**) and averaged over the corresponding emission angle intervals, respectively. The error bars were derived by error propagation from those of the single directions.

The anisotropic molecular potential influences the photoionisation dynamics, as demonstrated in Fig. 4a, b, which present the difference of the time delays measured along the directions A and C (Δ*τ*_*A*−*C*_ = *τ*_*A*_ − *τ*_*C*_) and B and C (Δ*τ*_*B*−*C*_ = *τ*_*B*_ − *τ*_*C*_), respectively. The experimental values (red squares) were determined integrating the photoelectrons emitted in the parallel configuration over the emission angles *θ* in the intervals *θ*_*A*_ = [0−29^∘^], *θ*_*B*_ = [60−75.5^∘^], and *θ*_*C*_ = [151−180^∘^] along the directions A, B, and C, respectively. The theoretical values (black circles) were obtained from the calculated RABBITT spectra in the same way. The delay difference Δ*τ*_*A*−*C*_ presents a minimum in the experimental as well as in the theoretical data around 34 eV photon energy, which approximately matches the maximum of the shape resonance for the direction C observed in Fig. 3c. For photon energies beyond the shape-resonance regions of both directions, the absolute value of the difference Δ*τ*_*A*−*C*_ becomes smaller.

The experimental points are in good agreement with the theoretical curve (except for the point corresponding to sideband 20). The stereo Wigner time delay^22,32,33^ obtained from the one-photon (XUV) dipole matrix elements and integrated over the corresponding angle intervals is also reported (blue triangles) and indicates a minimum in the same energy region. The deviations between the difference of the stereo Wigner time delays and the corresponding time delays obtained for the two-colour simulations, support the conclusion that the delay in photoionisation of molecules cannot be decomposed, in general, into the sum of a contribution due to the Wigner delay and one associated with the continuum-continuum delay^22^. The latter, indeed, should cancel out when subtracting the delay estimated along the directions A and C. In any case, the overall evolution of the delay is qualitatively similar suggesting the relevance of the differences in the position of the shape resonances along the directions A and C in the photoionization process. Differently from the previous case, the experimental data for the delay difference Δ*τ*_*B*−*C*_ (red square) do not show any significant variation of the delay as a function of the photon energy, as expected. The theoretical values (black circle) are in good agreement with the experimental data, indicating only a moderate linear increase of the delay. The absence of any remarkable variation of the delay as a function of the sidebands for these two directions can be attributed to the similar heights of the trapping potentials, as shown in Fig. 3b. In this condition, the evolution of the stereo Wigner time delays (blue triangles) is close to the experimental one.

We have demonstrated that the anisotropic molecular landscape affects the stereo-photoemission time delays. In particular, different heights of the trapping potentials introduce different delays in the emission of the photoelectron wave packet into the continuum. Our results indicate the importance of coincidence spectroscopy to disentangle the information on the photoemission process from different electronic states and for different molecular orientations. Extension of this approach would be beneficial also for larger and more complex molecular systems.

## Methods

### Experimental setup

The experiment was performed using a 10 kHz laser Ti:sapphire system providing 30-fs laser pulses centred at 800 nm with a pulse energy of 1 mJ. The input pulse first passes through a 1-mm-thick glass plate with a 3-mm-diameter hole at the centre splitting the beam into two parts (annular and central part), which are spatially separated and temporally delayed. Then, the input beam is focused by 25-cm-focal-length parabolic mirror into a high-harmonic gas cell filled with argon (Ar) or krypton (Kr) to generate XUV photons (20–46 eV) consisting of odd multiples of the input pulse frequency.

In our work, the XUV photons were generated by the annular beam. By using an iris before the gas cell and blocking the annular beam, we ensure that the central beam, after focusing it into the gas cell, is not contributing to the XUV emission. After the gas cell, another 1-mm-thick glass plate with a 1-mm-diameter hole, centred with respect to the first plate, is placed to synchronise the XUV and IR probe pulses. The IR probe pulse is part of the input beam which is going through the hole of the first plate and glass of the second one. The delay between XUV and IR probe pulse is varied precisely by tilting the second, drilled plate^26^. For the XUV-only measurements, an aluminium filter is introduced into the beam propagation direction after the high-order harmonic generation cell in order to block the co-propagating IR pulse. Then, the XUV and probe pulses are refocused by a toroidal mirror into the interaction region inside a 3D momentum imaging spectrometer leading to dissociative photoionisation of CF_4_ at an ion count rate of about 0.1 per XUV pulse. The resulting ions and electrons are guided using a homogeneous electric field (∣**E**∣ ~ 313 V/m) and a weak magnetic field (∣**B**∣ ~ 9.4 G) towards time- and position-sensitive detectors located at the opposite ends of the spectrometer.

### Theoretical methods

In brief, theoretical RABBITT spectra have been obtained by solving the time-dependent Schrödinger equation (TDSE) within the static-exchange approximation^15,31^. In this method, the time-dependent wave function is expanded in a basis set of *N*-electron stationary wave functions, built from antisymmetrized products of an (*N* − 1)-electron wave function for the bound electrons and a one-electron continuum wave function for the electron ejected into the continuum. The (*N* − 1)electron wave functions are built from B-spline representations of the lowest Kohn-Sham (KS) orbitals resulting from the diagonalization of the KS hamiltonian of density functional theory, excluding the orbital from which the electron is ejected to the continuum, and the one-electron continuum wave functions are obtained from an inverse iterative procedure in the former KS orbital basis for each photoelectron energy. All dipole matrix elements describing the transitions between all bound and continuum states as well as between continuum states have been included in the solution of the TDSE, which is essential to describe the bound-continuum and continuum–continuum transitions leading to the RABBITT spectra. We have restricted all simulations to the equilibrium geometry (fixed-nuclei approximation). More details can be found in the Supplementary Information.

## Supplementary information

Suppelementary Information

## Data Availability

The data that support the findings of this study are available on reasonable request from the corresponding author G.S.(giuseppe.sansone@physik.uni-freiburg.de). Requests for data will be processed within 1 week. The data are not publicly available due to further analysis on the findings conducted by the authors of this manuscript.

## Code availability

Analysis codes used in this study are available on reasonable request from the corresponding author G.S.(giuseppe.sansone@physik.uni-freiburg.de). Requests for codes will be processed within one week.
