# Supplementary material for: Attosecond photoionisation time delays reveal the anisotropy of the molecular potential in the recoil frame
Source: Nat Commun. 2022 Mar 10;13:1242. doi: 10.1038/s41467-022-28783-x (PMC8913798; doi:10.1038/s41467-022-28783-x)
Supplement: Supplementary file 1 — Suppelementary Information [file 41467_2022_28783_MOESM1_ESM.pdf]

Supplementary Information

Attosecond photoionisation time delays reveal the anisotropy of the molecular potential in the recoil frame.

H. Ahmadi*,[1,2] E. Plésiat*,[3,4] M. Moioli,[1] F. Frassetto,[5] L. Poletto,[5] P. Decleva,[6] C .D. Schröter,[7] T. Pfeifer,[7] R. Moshammer,[7] A. Palacios,[3,8] F. Martin†,[3] and G. Sansone†[1]

[1]*Physikalisches Institut, Albert-Ludwigs-Universität*
Stefan-Meier-Straße 19, 79104 Freiburg, Germany

[2]*Dipartimento di Fisica, Politecnico di Milano*
Piazza Leonardo da Vinci 32, 20133 Milano Italy

[3]*Universidad Autónoma de Madrid,*
Facultad de Ciencias Cantoblanco Madrid 28049 Spain

[4]*Instituto Madrileño de Estudios Avanzados en Nanociencia*
(IMDEA-Nanociencia), Cantoblanco, 28049 Madrid, Spain

[5]*CNR-IFN Padua, Italy*

[6]*CNR IOM and Universitá di Trieste, 34127 Trieste, Italy*

[7]*Max-Planck-Institut für Kernphysik, 69117 Heidelberg, Germany*

[8]*Institute for Advanced Research in Chemical Sciences (IAdChem),*
Universidad Autónoma de Madrid, 28049 Madrid, Spain

* *These authors contributed equally to this work*

† *Corresponding authors: fernando.martin@uam.es;*
giuseppe.sansone@physik-uni.freiburg.de

Data acquisition and data analysis

Extreme-ultraviolet spectroscopy of CF_4

Photons with energies between 20-46 eV were generated in krypton and argon (see Supplementary Fig. 1). The extreme ultraviolet (XUV) spectra can ionise CF_4 from one of its five valence molecular orbitals, leaving the parent ion CF_4^+ in its X^2T_1, A^2T_2, B^2E, C^2T_2 and D^2T_2 states. The ground and excited states of CF_4^+ are unstable and dissociate through different pathways into CF_3^+ and CF_2^+ ions. The typical time-of-flight (TOF) spectrum generated by the interaction with only the XUV pulse is shown in Supplementary Fig. 2.

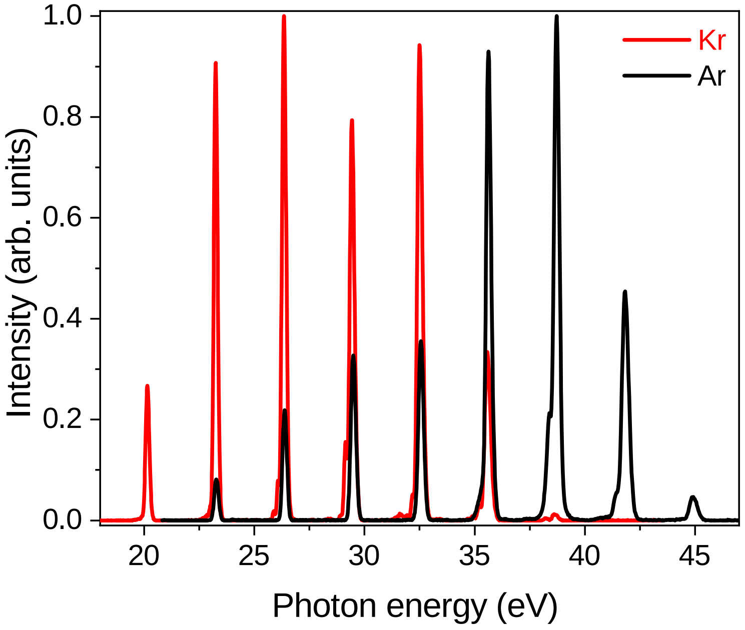

Supplementary Fig. 1: Experimental XUV spectra. Intensity profiles of the XUV spectra generated in Ar (black) and Kr (red) and used in the experiments.

The two main peaks correspond to CF_2^+ (green) and CF_3^+ (blue) fragments. The small peak around 13 μs has been assigned to H_2O^+.

The conventional photoelectron-photoion coincidence measurements have established the relative contribution of these five states into different fragments as presented in Supplementary Table 1 [1, 2]. The mean kinetic energy release (KER) for photoionisation from these three states are also reported in Supplementary Table 1. The lowest three states X^2T_1, A^2T_2 and B^2E dissociate exclusively through the CF_3^++ F channel [3], corresponding to a branch-

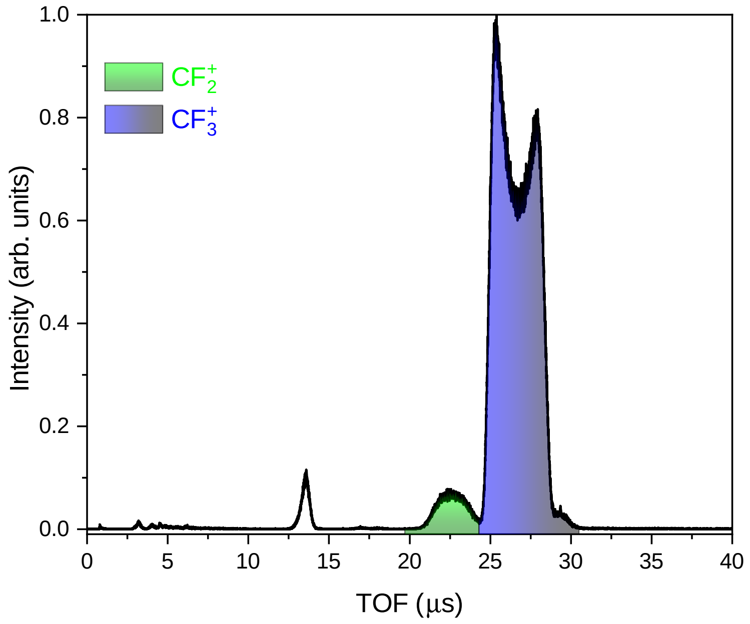

Supplementary Fig. 2: TOF ion spectra. TOF ion spectra showing the two dominant fragments (CF_2^+ in green and CF_3^+ in blue) after photoionisation by the XUV harmonic spectra.

ing ratio of 100% in this channel. The C^2T_2 state dominantly fragments into CF_2^+ [3, 4] (branching ratio of 62%), while D^2T_2 state dissociates into CF_3^+ and CF_2^+ according to ref. [3] and into CF^+ according to ref. [1]. Fragmentation of the D^2T_2 state into CF_3^+ and CF_2^+ can be ignored because of relatively strong contribution of the other states.

To illustrate the procedure for isolating the contribution of photoionisation from the X^2T_1 ground-state, we report in panels (a) of Supplementary Fig. 3 and Supplementary Fig. 4, the KER as a function of the energy of the photoelectron measured in coincidence with the fragment CF_3^+ using the XUV harmonics generated in krypton and argon, respectively. For KERs comprised between 0 and 1 eV, the photoelectron spectra are characterised by a well defined harmonic structure, as shown in the panels (b). This is consistent with the observation that the mean kinetic energy of only the ground state X^2T_1 is below 1 eV (see Supplementary Table 1). For higher KERs, additional peaks appear between the main ones (in particular at low photoelectron energies) as revealed by the photoelectron spectrum integrated over the range 0-3 eV (panels (b)). These additional contributions are attributed to photoelectrons released after photoionisation from the A^2T_2, B^2E, and C^2T_2 states of CF_4, which leads to the emission of CF_3^+ fragments with higher KERs, in agreement with

Supplementary Table 1: Energies, states and branching ratios for the photoionisation and photodissociation of CF_4. Ionization energy (IE), fragment ion, branching ratio and average kinetic energy release from dissociation of states of CF_4^+. The values are derived from refs. [1, 2]. The branching ratios for the D^2A_1 state are not indicated, as the fragments CF_3^+ and CF_2^+ were not associated to the photoionisation from this state in ref. [1].

State	IE (eV)	Fragment ion	Branching ratio	<KER> (eV)
X^2T_1	16.3	CF_3^+	100%	0.97±0.05
A^2T_2	17.5	CF_3^+	100%	1.19±0.04
B^2E	18.3	CF_3^+	100%	1.27±0.14
C^2T_2	22.2	CF_3^+	38%	1.34±0.10
C^2T_2	22.5	CF_2^+	62%	0.57±0.06
D^2A_1	25.3	CF_3^+	–	1.54±0.13
D^2A_1	25.3	CF_2^+	–	1.50±0.26

the values reported in Supplementary Table 1. In order to isolate the contribution of the photoelectrons originating from the ground-state of CF_4, only those electrons measured in coincidence with a CF_3^+, with KER lower than 1 eV were considered.

Delay extraction

The measurements are performed using the RABBITT (reconstruction of attosecond beating by interference of two-photon transitions) technique [5], which is based on single-photon ionisation in which trains of attosecond pulses ionise the system and a synchronised IR pulse interacts with the ionised electron. Photoelectron kinetic energy is recorded as a function of delay τ between the XUV-pump and IR-probe pulses. The XUV pulse consists of odd multiples of the fundamental frequency ω_{IR} of the IR probing laser (λ_{IR} =800 nm). Therefore, photoelectron spectrum shows discrete peaks by absorbing one XUV photon, whose energy is higher than the ionisation potential of the system. The subsequent absorption of one IR photon leads to sidebands (Sb) which appears between the odd harmonics. For each sideband order $2q$, two indistinguishable excitation pathways are involved: (1) ab-

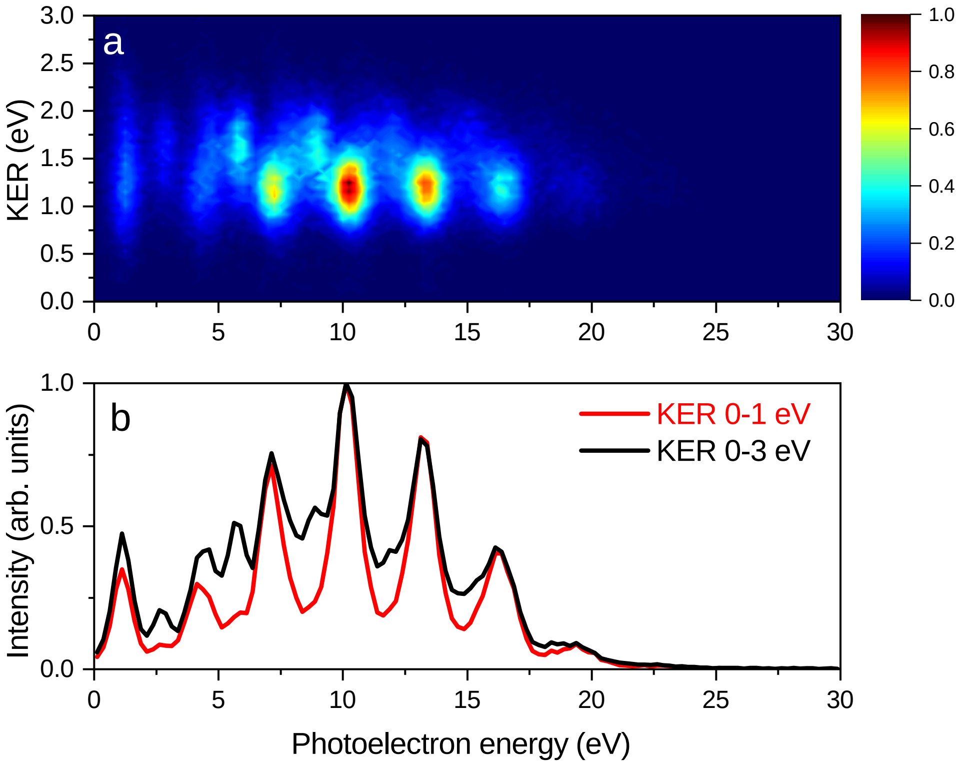

Supplementary Fig. 3: KER and photoelectron energies for XUV spectra generated in krypton. a) KER as a function of the energy of the photoelectrons measured in coincidence with CF_3^+ ions measured using the HHG spectrum generated in krypton. (b) Photoelectron spectrum obtained by integrating over two KER ranges: 0-1 eV (red curve) and 0-3 eV (black curve).

sorption of one photon from harmonic $2q-1$ followed by the absorption of an additional IR photon, and (2) absorption of one photon from harmonic $2q+1$ and subsequent emission of an IR photon. Because of two-quantum path interference, the sideband intensity shows an oscillation by varying the time delay (τ) : $S(\mathbf{p},\tau) \propto a + b\cos(2\omega_{\mathrm{IR}}\tau - \varphi_{\mathrm{atto}} - \varphi_{\mathrm{tot}})$, where a and b are parameters and $\mathbf{p}$ is the momentum of the photoelectron. The terms φ_{atto} and φ_{tot} are related to the chirp of the XUV pulse train and the phase introduced by the photoionisation process in the two-photon transition from the initial to the final state. Therefore, the attosecond time delay estimated from the RABBITT traces contains a contribution related to the attosecond chirp and one due to the photoionisation process:

$$\tau_{\mathrm{RAB}} = \tau_{\mathrm{atto}} + \tau_{\mathrm{tot}} = \frac{\varphi_{\mathrm{atto}}}{2\omega_{\mathrm{IR}}} + \frac{\varphi_{\mathrm{tot}}}{2\omega_{\mathrm{IR}}} \tag{1}$$

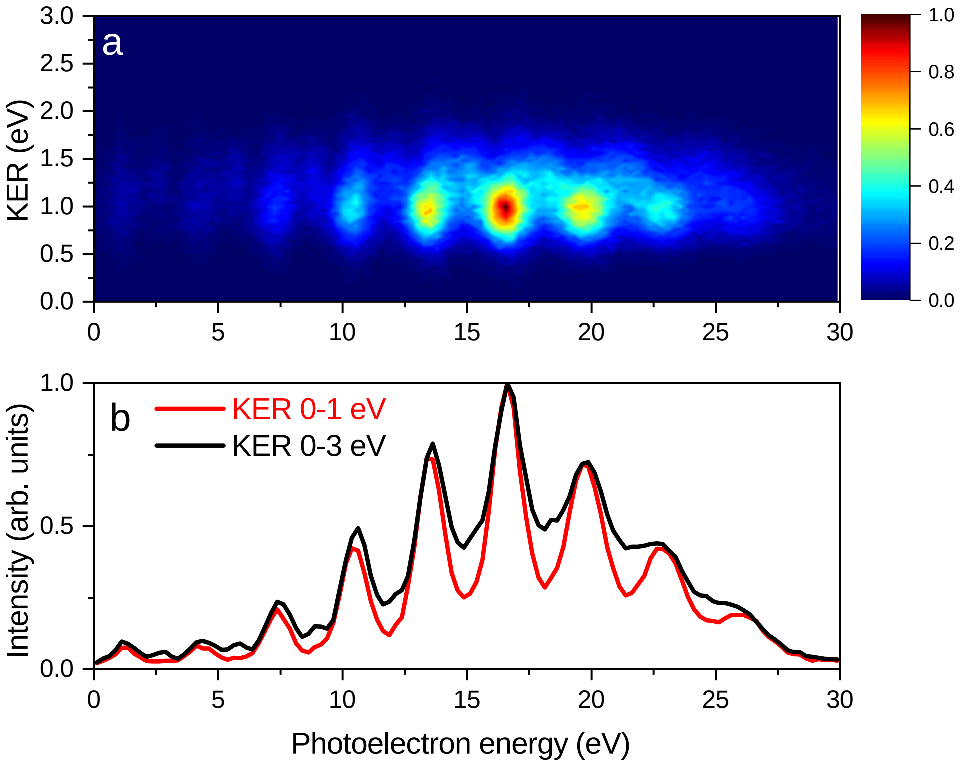

Supplementary Fig. 4: KER and photoelectron energies for XUV spectra generated in argon. a) KER as a function of the energy of the photoelectrons measured in coincidence with CF_3^+ ions measured using the HHG spectrum generated in argon. (b) Photoelectron spectrum obtained integrating over two KER ranges: 0-1 eV (red curve) and 0-3 eV (black curve).

To extract the delays from the measured data, a Fourier transform is performed along the delay axis to identify the component oscillating with $2\omega_{\mathrm{IR}}$. To obtain the error bars of the attosecond time delays, the results were weighted with the root-mean-square phase noise over an integrated electron kinetic-energy range of 1.2 eV centered around the maximum of each sideband [6].

To eliminate the time delay related to the attosecond chirp, we evaluate the delay extracted from the RABBITT traces for the different sidebands averaging over the photoemission angle for the parallel and perpendicular cases. The results, shown in Supplementary Fig. 5, indicate a monotonic increase of the delay for harmonic generated both in krypton and in argon, which can be attributed to the chirp of the attosecond pulses. The differences of the delays for the parallel and perpendicular cases are within the error bars for each of

the two generation gases.

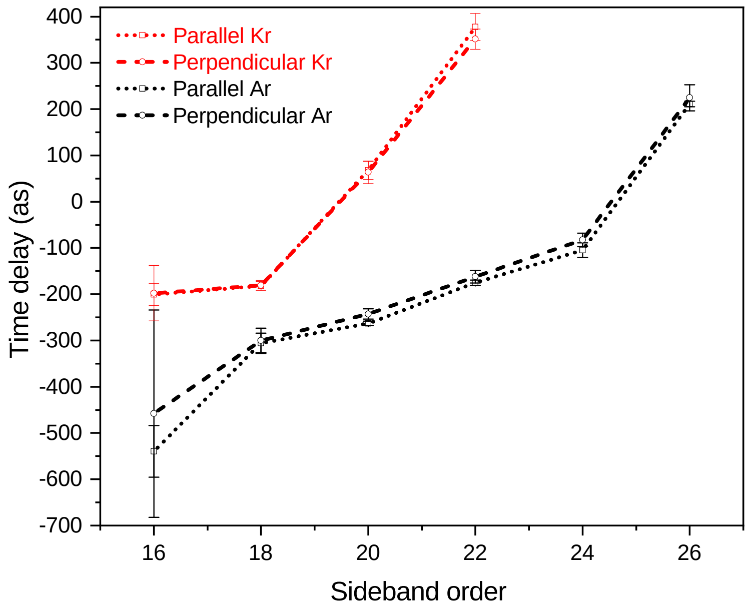

Supplementary Fig. 5: Attosecond time delays averaged over the emission directions. Measured attosecond time delays averaged over the photolectron emission directions for the parallel (dotted lines, square) and perpendicular (dashed lines, circle) cases for the measurement acquired in Kr (red) and Ar (black). See text for the definition of the error bars.

The error bars presented in Figs. 2,4 of the main manuscript and in Supplementary Figs. 9, 10 are obtained using the same procedure outlined above, taking into account also the error bar in the estimation of the attochirp (τ_{atto}).

Theory and simulations

Theoretical model

The theoretical RABBITT spectra have been obtained by solving the TDSE within the static-exchange method employed in previous works [7, 8]. We restrict the simulations to the equilibrium geometry.

Within these approximations and assuming that the electronic Hamiltonian can be written as a sum of single-electron Hamiltonians, the time evolution of the system is obtained by solving the single-particle time-dependent Schrödinger equation:

$$\left[\hat{h}_{KS}^{(j)} + \hat{v}^{(j)}(t)\right] \psi_k \left(\mathbf{r}_{q_j}, t\right) = i \frac{\partial \psi_k \left(\mathbf{r}_{q_j}, t\right)}{\partial t}, \tag{2}$$

where $\hat{h}_{KS}^{(j)}$ is the Kohn-Sham (KS) hamiltonian describing the ground state of the molecule and $\hat{v}^{(j)}(t) = \mathbf{E}(t) \cdot \mathbf{r}_j$ is the interaction potential in the length gauge, with $\mathbf{E}$ the electric field of the light pulses (in the length gauge). $\psi_k \left(\mathbf{r}_{q_j}, t\right)$ is the time-dependent one-electron wave function for an electron j and an initial orbital k, written as a linear combination of field-free KS orbitals:

$$\psi_k \left(\mathbf{r}_{q_j}, t\right) = \sum_n c_{kn}(t) \varphi_n \left(\mathbf{r}_{q_j}\right) e^{iE_n t} + \sum_{q,l,\varepsilon} c_{kql\varepsilon}(t) \varphi_{ql\varepsilon} \left(\mathbf{r}_{q_j}\right) e^{i\varepsilon t}, \tag{3}$$

$\{\varphi_n \left(\mathbf{r}_{q_j}\right)\}$ corresponds to the bound stationary states with energy E_n and $\{\varphi_{ql\varepsilon} \left(\mathbf{r}_{q_j}\right)\}$ to the discretized continuum stationary states of symmetry q, angular momentum l and photoelectron energy ε. The initial conditions are imposed by setting all coefficients to zero except $c_{kk} = 1$.

Bound and continuum stationary states are obtained by solving the KS equations with the LB94 exchange-correlation functional [9]. The electronic density is first calculated with the Amsterdam Density Functional package with a DZP basis set. This electronic density is used to build and diagonalize the field-free KS Hamiltonian in a multicenter basis set of B-splines functions and symmetry-adapted real spherical harmonics [10]. The B-spline basis set contains a large number of functions located at the center of mass of the molecule (denoted 0) and a smaller number centered on the fluorine atoms (denoted 1). The radii and the maximum angular momenta of the expansion centers are respectively R_{max}^0 =800

a.u., R^1_{max} =0.85 a.u. and l^0_{max} =14, l^1_{max} =2.

The angularly resolved ionisation probability for a specific photoionisation channel and molecular orientation is given by:

$$\frac{\partial P}{\partial\varepsilon\partial\Omega_e} = \left|\left\langle\varphi^{(-)}(\hat{\mathbf{k}}) \mid \psi_k\left(\mathbf{r}_{q_j}, t = T_{max}\right)\right\rangle\right|^2 = \left|\sum_{q,l}(-i)^l e^{i\sigma_l(\varepsilon)} X_{ql}(\Omega_e) T^{(-)}_{\varepsilon ql}\right|^2 \tag{4}$$

with

$$T^{(-)}_{\varepsilon ql} = \left\langle\varphi^{(-)}_{\varepsilon ql} \mid \psi_k\left(\mathbf{r}_{q_j}, t = T_{max}\right)\right\rangle \tag{5}$$

where T_{max} is larger than or equal to the total duration of the XUV and IR pulses and $\varphi^{(-)}_{\varepsilon ql}$ are the continuum states obtained with the Galerkin approach [11]. The superscript $^-$ denotes the correct incoming scattering boundary condition. $\sigma_l(\omega)$ corresponds to the Coulomb phase shift and $X_{ql}(\Omega_e)$ are the symmetry adapted real spherical harmonics defined at the center of mass of the molecule:

$$X_{ql}(\Omega_e) = \sum_m b_{qlm} Y_{lm} \tag{6}$$

In order to reproduce the experimental conditions, we have employed an attosecond pulse train with a total duration of 20 fs, with 14 single attosecond pulses, together with a time-delayed IR 20 fs pulse. We have checked convergence with different power intensities of the pulses to ensure we are working within the perturbative regime, while still obtaining a significant ionisation signal in the sidebands. The present calculations have been obtained with intensities of 4×10^{10} and 2×10^{11} W/cm^2, for the XUV and the IR fields, respectively. These simulations assume linearly polarized light interacting with the molecule in different orientations defined by the angles β and α (see Fig. 1 of the main manuscript).

Cross-sections and recoil frame photoelectron angular distributions: comparison between theory and experiment

Supplementary Fig. 6(a) presents a comparison between the experimental total XUV photoelectron spectra (black curve) and the theoretical result obtained solving the TDSE with the theoretical attosecond pulse train. The experimental total photoelectron spectrum is retrieved by collecting all photoelectrons measured in the photoionisation of CF_4. In the

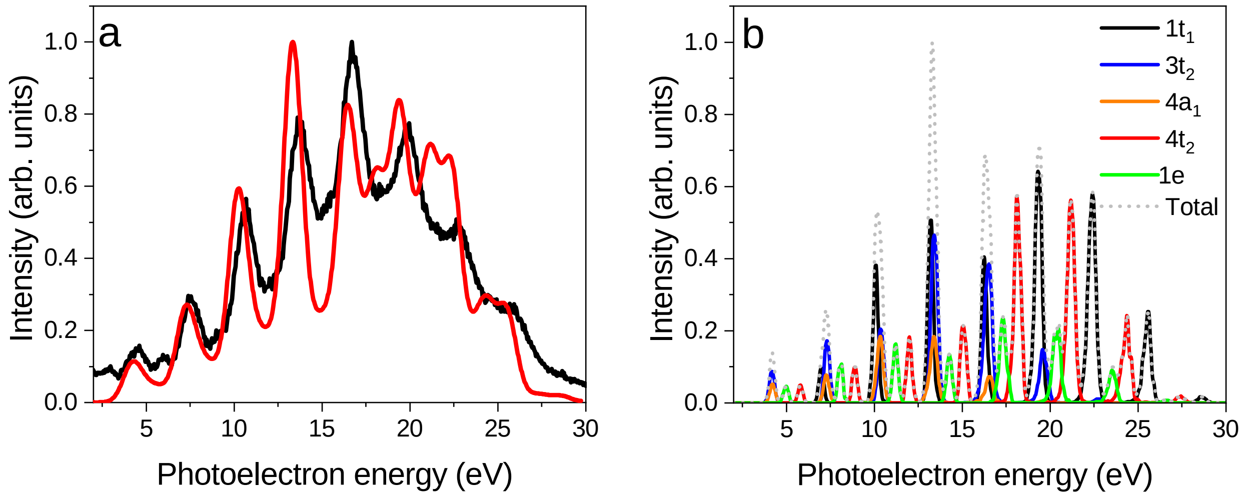

Supplementary Fig. 6: Experimental and channel-resolved simulated cross-sections. a) Experimental (black) and theoretical (red) XUV-only photoelectron spectra averaged over electron and molecular angles. The theoretical spectrum has been convoluted with the experimental energy resolution. b) Contributions of each photoionization channel to the total theoretical XUV spectrum (gray dotted curve).

simulation, photoelectron spectrum of each five valence states of CF_4 is first calculated and then added up in order to compare with the experimental spectrum.

The red curve corresponds to the convolution using a Gaussian window with a $\sigma = 1.5$ eV, in order to take into account the finite experimental energy resolution. As shown in the figure, the theoretical spectrum is in a good agreement with the experimental one. The contribution of the five different photoionisation channels to the total photoelectron spectra is presented in Supplementary Fig. 6(b). In both figures, the theoretical (KSLB94) IPs have been corrected (post-TDSE) to match the experimental values (see Table 1). The comparison between the experimental results and theoretical predictions for the recoil frame photoelectron angular distributions (RFPADs) generated by the attosecond pulse train only for the parallel and perpendicular configurations is shown in Supplementary Fig. 7 and Supplementary Fig. 8, respectively. The overall agreement is good. In particular, both experimental and theoretical RFPADs present a strong asymmetry as a function of θ for the parallel case.

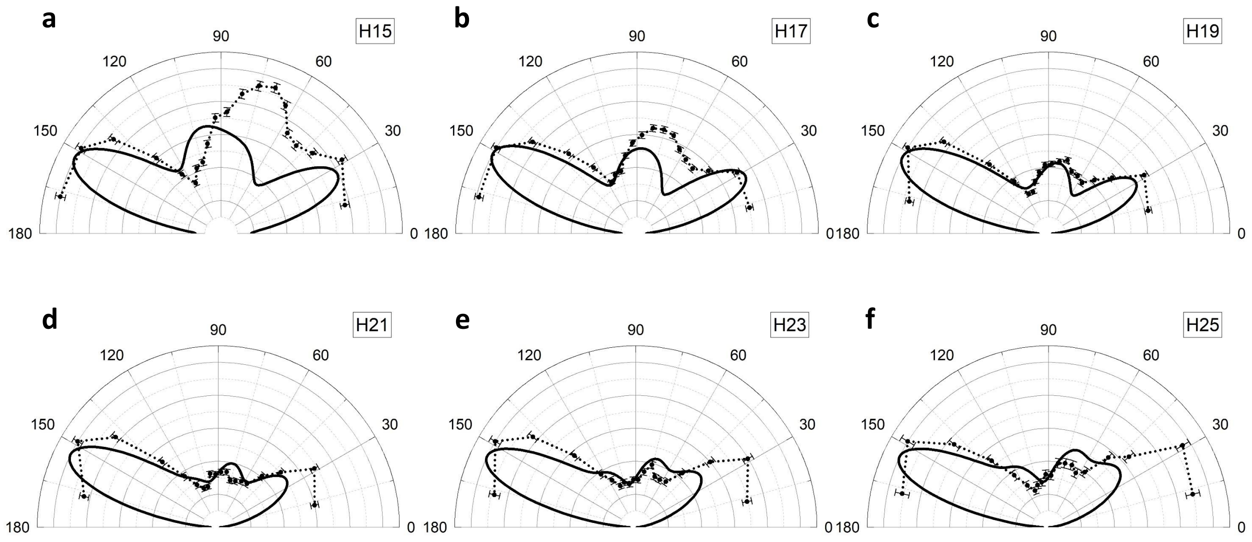

Supplementary Fig. 7: Experimental and simulated RFPADs for the parallel case. Comparison between XUV-only experimental (black circles and dotted line) and theoretical (solid line) RFPADs for the parallel orientation for the harmonic orders from H15 to H25 (panels from a to f). The error bars are estimated as the square root of the total number of counts at that specific angle range.

Recoil-frame attosecond time delays

Supplementary Figs. 9 and 10 present the complete dataset for the dependence of the time delays on the emission angle θ for the parallel and perpendicular configurations, respectively. The time delay for the Sb16-18-20 and for the Sb22-24-26 were extracted from the photoelectron spectra obtained using attosecond pulse train generated in krypton and argon, respectively. We considered photoelectrons emitted between 0° to 65° degrees with respect to the polarisation axis in the laboratory frame. The attosecond chirp was removed by subtracting the emission-angle-averaged time delay for the parallel and perpendicular cases presented in Supplementary Fig. 5. For the parallel case, the recoil-frame attosecond time delays present a minimum around $\theta = 90°$, while no significant structure can be observed for the perpendicular case. This evolution is well-reproduced by the simulations.

Supplementary Fig. 11 presents the evolution of the attosecond time delays for different

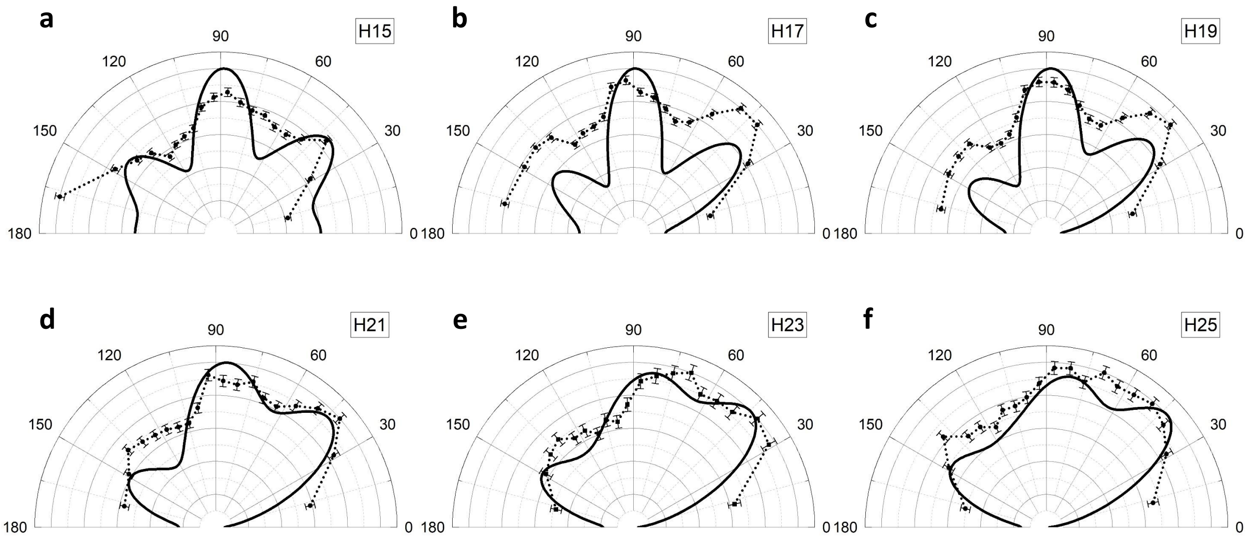

Supplementary Fig. 8: Experimental and simulated RFPADs for the perpendicular case. Comparison between XUV-only experimental (black circles and dotted line) and theoretical (solid line) RFPADs for the perpendicular orientation for harmonic orders from H15 to H25 (panels from a to f). The error bars are estimated as the square root of the total number of counts at that specific angle range.

molecular orientations ($\beta = 0°, 15°, 30°, 60°, 90°, 120°$; $\alpha = 0°$). Time delays for Sb 18-26 present a variation corresponding to a semi-optical cycle at small β angles. The large variation (reported also Fig. 12 (a,b,c) for Sb18 and Sb20) in is absent in the Wigner time delays calculated using the photoionisation matrix element (see Supplementary Fig. 12 (d,e,f)).

For comparison with the experiment, we have integrated over cones of the polar angle β: 0-30° for the parallel and 60-120° for the perpendicular orientations. As shown in Supplementary Fig. 13, the time delays do not depend strongly on the azimuthal angle α which has been fixed to 0° in all the RABBITT theoretical calculations. In the case of the one-photon Wigner delays shown in Fig. 4 of the main manuscript, the derivative of the phase of the corresponding dipole couplings has been averaged over the photoelectron emission angle φ, the molecular angle α, and an ion cone of $0 - 45°$ of the molecular angle β using a similar procedure as the one presented in Eq. 6 of [12].

The presence of the minimum can be attributed to an interplay between the ground state

of the HOMO orbital (well-approximated by 2p orbitals centered on the four fluorine atoms), the angular dependence of the effect of the IR field, and the averaging over the angle β defining the relative orientation of one CF-bond and the electric field. The simulation shows that for increasing angles β, the effect of the IR field leads to a minimum of the delay at $\theta = 90°$. As the averaging over the molecular axis is dominated by the contribution at larger angles due to the solid-angle element, the delay as a function of the emission angle of the photoelectron presents a minimum as observed in the experiment. From the simulation, it can be observed that the transition occurs in a limited range going from $\theta \approx 70°$ to $\theta \approx 110°$.

For the perpendicular case, the dissociation occurs in the direction perpendicular to the polarisation direction of the field. The averaging effect over the molecular axis leads to a flat evolution of the delay as a function of the emission angle in the recoil frame. The largest variations of the delay are observed around $\theta = 0°$ and $180°$ in the simulations, i.e. for an emission angle perpendicular to the IR field polarization. As shown in Supplementary Fig. 8, the cross-section is very small for angles close to $\theta = 0°$ and $180°$. As a consequence, the counts of photoelectrons is too low to retrieve the time delays from the experimental data in these regions of angles.

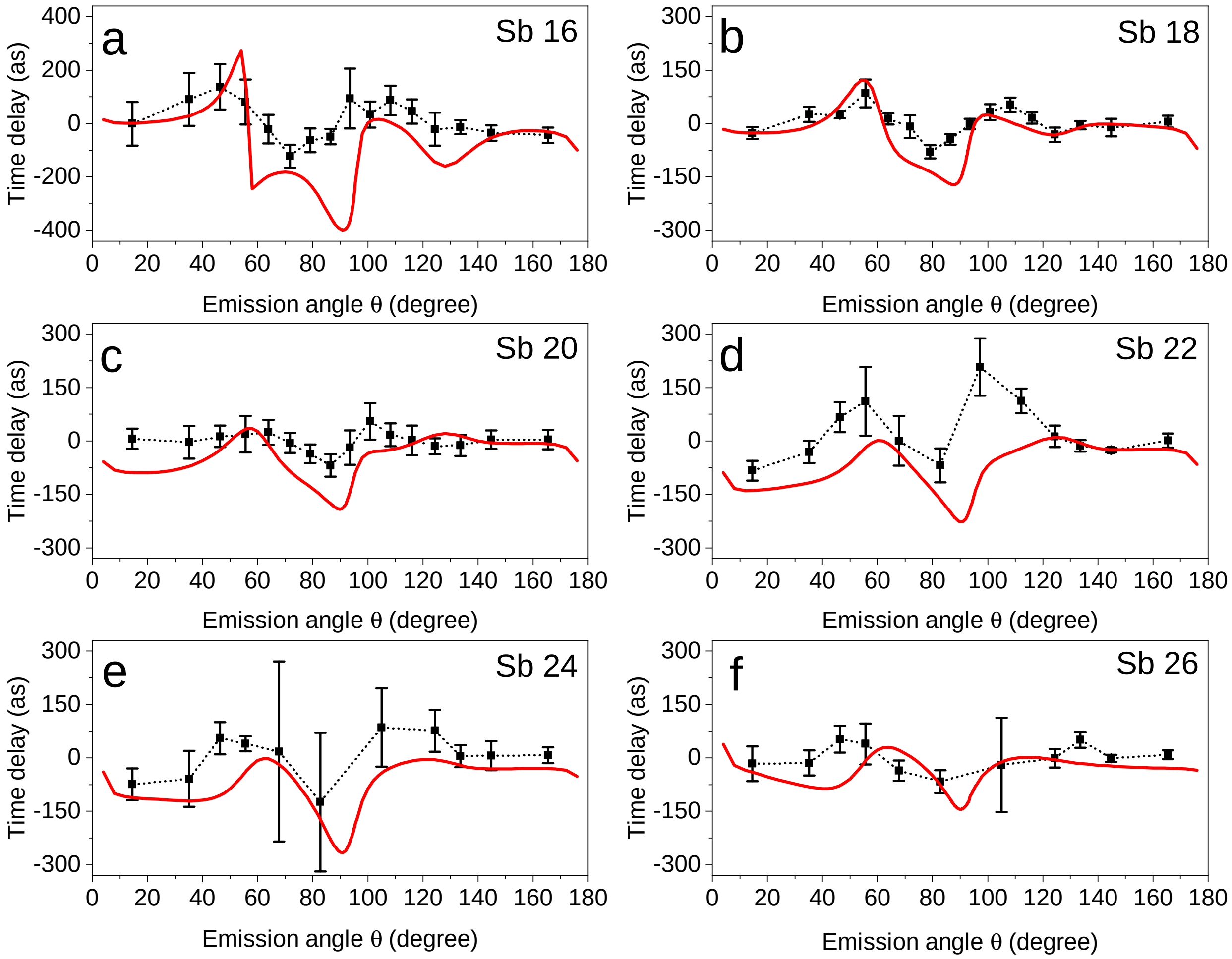

Supplementary Fig. 9: Experimental and simulated attosecond time delays in the recoil-frame for the parallel case. Evolution of the attosecond time delays extracted from the experimental (black) and theoretical (red) RABBITT traces for the sideband orders Sb16-Sb26 (panels a-f) for the parallel case. See text for the definition of the error bars.

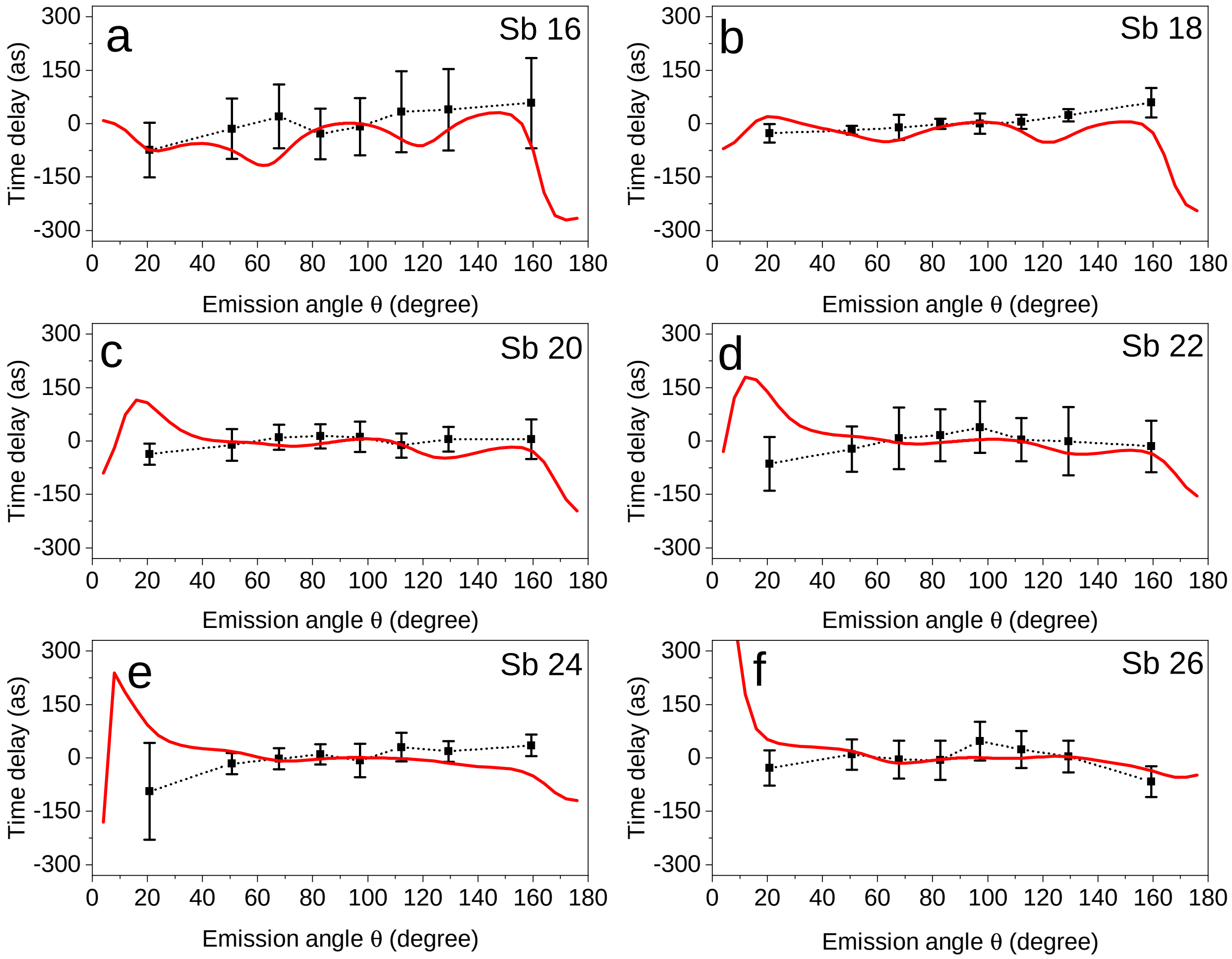

Supplementary Fig. 10: Experimental and simulated attosecond time delays in the recoil-frame for the perpendicular case. Evolution of the attosecond time delays extracted from the experimental (black) and theoretical (red) RABBITT traces for the sideband orders Sb16-Sb26 (panels a-f) for the perpendicular case. See text for the definition of the error bars.

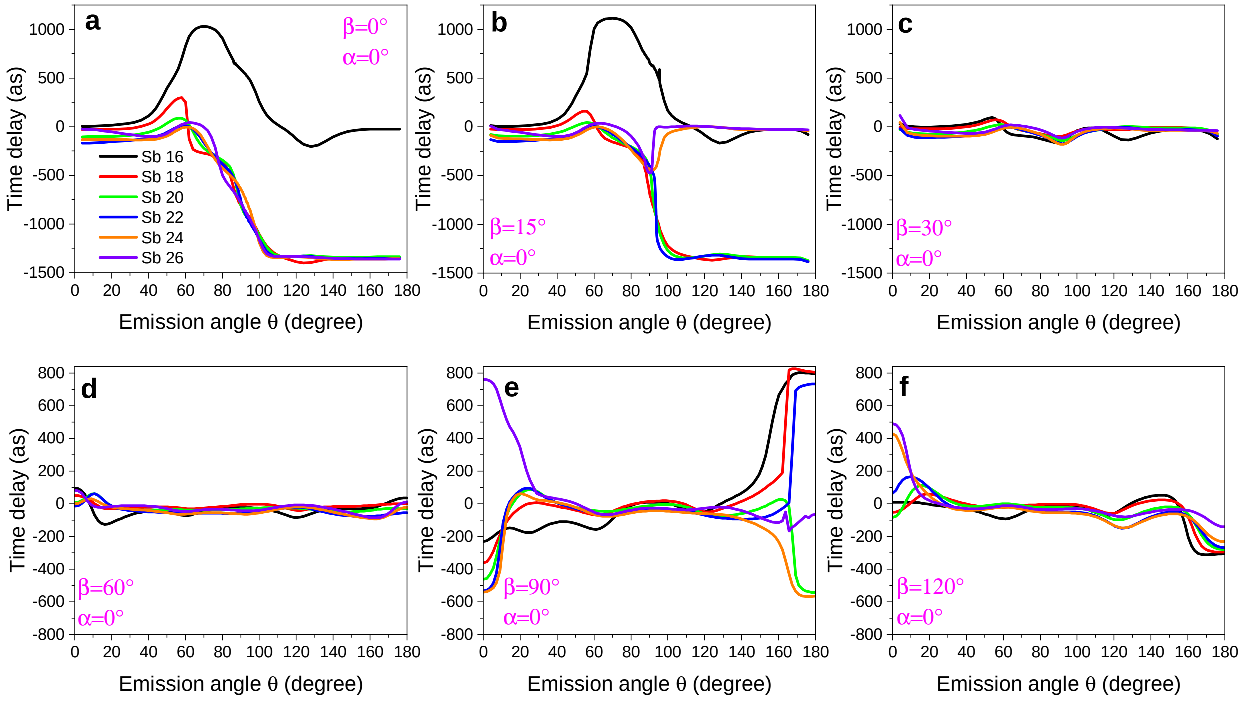

Supplementary Fig. 11: Simulated attosecond time delays for different angles β. Evolution of the attosecond time delays extracted from the simulated RABBITT traces for the sideband orders Sb16-Sb26 and for different values of the angle β, for the parallel (a,b,c) and perpendicular case (d,e,f) cases. The values of the angles of the electric field direction β and α are indicated in the panels.

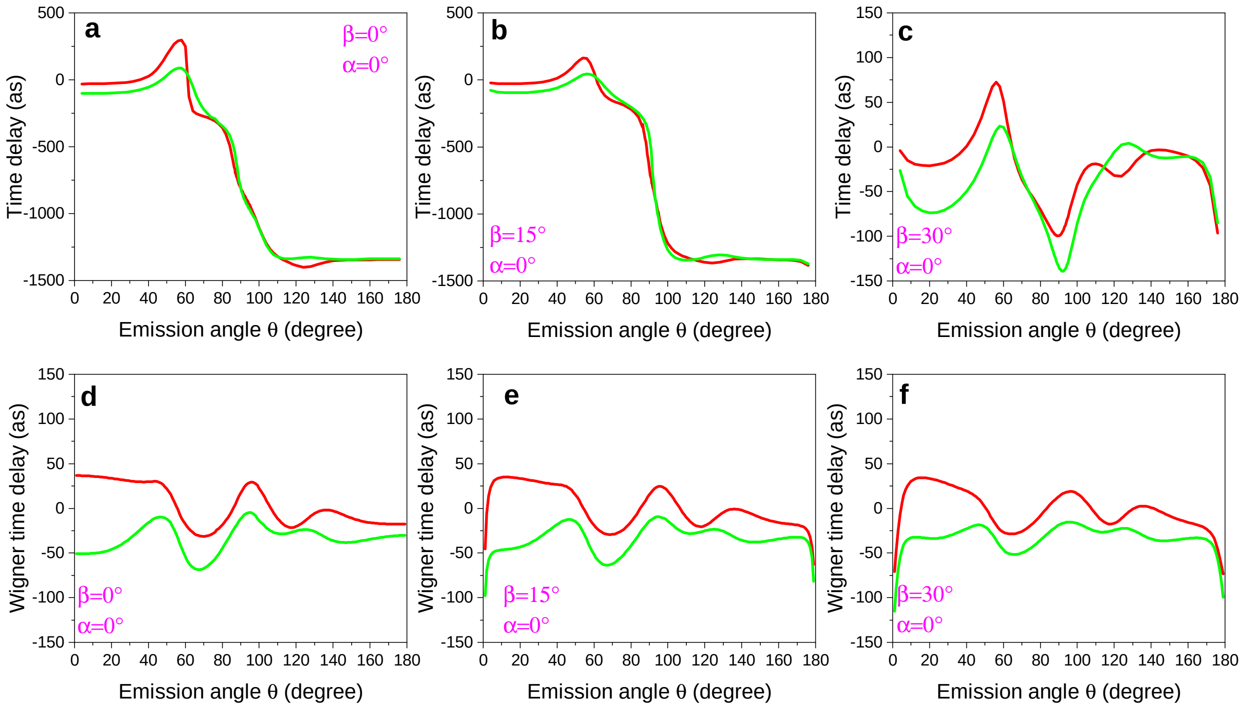

Supplementary Fig. 12: Simulated Wigner and attosecond time delays for different angles β**.** Comparison of the simulated attosecond (a,b,c) and Wigner (d,e,f) time delays for the sideband orders Sb18 (red) and Sb20 (green) for the different angle β: 0° (a,d), 15° (b,e), and 30° (c,f).

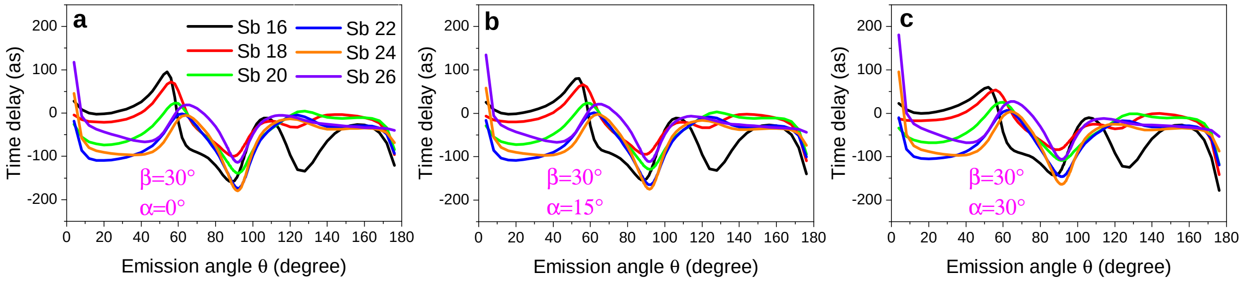

Supplementary Fig. 13: Simulated attosecond time delays for different angles α**.** Evolution of the attosecond time delays extracted from the simulated RABBITT traces for the sideband orders Sb16-Sb26 and for β=30° and different values of the angle α: 0° (a), 15° (b), and 30° (c). (see Fig. 1 for the definition of the angle) for the parallel case.

SUPPLEMENTARY REFERENCES

[1] Zhang, W., Cooper, G., Ibuki, T. & Brion, C.E. Excitation and ionization of freon molecules. I. Absolute oscillator strengths for the photoabsorption (12–740 eV) and the ionic photofragmentation (15–80 eV) of CF_4. *Chem. Phys.* **137**, 391-405 (1989).

[2] Creasey, J. C. et al. Fragmentation of Valence Electronic States of CF_4^+ and SF_6^+ Studied by Threshold Photoelectron Photoion Coincidence Spectroscopy. *Chem. Phys.* **174**, 441-452 (1993).

[3] Masuoka, T. & Kobayashi, A. Dissociative photoionization of CF_4 from 23 to 120 eV. *J. Chem. Phys.* **113**, 1559-1565 (2000).

[4] Creasey, J. C. et al. Nonradiative Decay Pathways of Electronic States of Group-IV Tetrafluoro and Tetrachloro Molecular-Ions Studied with Synchrotron Radiation. *J. Chem. Phys.* **93**, 3295-3306 (1990).

[5] Paul, P. M. et al. Observation of a Train of Attosecond Pulses from High Harmonic Generation. *Science* **292**, 1689-1692 (2001).

[6] Huppert, M. et al. Attosecond Delays in Molecular Photoionization. *Phys. Rev. Lett.* **117**, 093001 (2016).

[7] Plésiat, E. et al. Real-Time Imaging of Ultrafast Charge Dynamics in Tetrafluoromethane from Attosecond Pump-Probe Photoelectron Spectroscopy. *Chem. Eur. J.* **24**, 12061-12070 (2018).

[8] Nandi, S. et al. Attosecond timing of electron emission from a molecular shape resonance. *Sci. Adv.* **6**, eaba7762 (2020).

[9] van Leeuwen, R. & Baerends, E. J. Exchange-correlation potential with correct symptotic behavior. *Phys. Rev. A* **49**, 2421 (1994).

[10] Toffoli, D. et al. Convergence of the multicenter B-spline DFT approach for the continuum. *Chem. Phys.* **276**, 25-43 (2002).

[11] Stener, M. et al. Recent advances in molecular photoionization by density functional theory based approaches. *Theor. Chem. Acc.* **117**, 943-956 (2006).

[12] Baykusheva, D. & Wörner, H. J. Theory of attosecond delays in molecular photoionization.

Journ. Chem. Phys. **146**, 124306 (2017).
